# Targeted metabolome analysis reveals accumulation of metabolites in testa of four peanut germplasms

**DOI:** 10.3389/fpls.2022.992124

**Published:** 2022-09-16

**Authors:** Kun Zhang, Jing Ma, Sunil S. Gangurde, Lei Hou, Han Xia, Nana Li, Jiaowen Pan, Ruizheng Tian, Huailing Huang, Xingjun Wang, Yindong Zhang, Chuanzhi Zhao

**Affiliations:** ^1^College of Tropical Crops, Hainan University, Haikou, China; ^2^Institute of Crop Germplasm Resources (Institute of Biotechnology), Shandong Academy of Agricultural Sciences, Shandong Provincial Key Laboratory of Crop Genetic Improvement, Ecology and Physiology, Jinan, China; ^3^College of Agricultural Science and Technology, Shandong Agriculture and Engineering University, Jinan, China; ^4^College of Life Sciences, Shandong Normal University, Jinan, China; ^5^Crop Protection and Management Research Unit, USDA-ARS, Tifton, GA, United States; ^6^Department of Plant Pathology, University of Georgia, Tifton, GA, United States; ^7^Hainan Academy of Agricultural Sciences, Haikou, China

**Keywords:** testa color, flavonoids, LC-MS/MS, metabolome profiling, metabolic pathway

## Abstract

Cultivated peanut (*Arachis hypogaea* L.) is an important source of edible oil and protein. Peanut testa (seed coat) provides protection for seeds and serves as a carrier for diversity metabolites necessary for human health. There is significant diversity available for testa color in peanut germplasms. However, the kinds and type of metabolites in peanut testa has not been comprehensively investigated. In this study, we performed metabolite profiling using UPLC-MS/MS for four peanut germplasm lines with different testa colors, including pink, purple, red, and white. A total of 85 metabolites were identified in four peanuts. Comparative metabolomics analysis identified 78 differentially accumulated metabolites (DAMs). Some metabolites showed significant correlation with other metabolites. For instance, proanthocyanidins were positively correlated with cyanidin 3-O-rutinoside and malvin, and negatively correlated with pelargonidin-3-glucoside. We observed that the total proanthocyanidins are most abundant in pink peanut variety WH10. The red testa accumulated more isoflavones, flavonols and anthocyanidins compared with that in pink testa. These results provided valuable information about differential accumulation of metabolites in testa with different color, which are helpful for further investigation of the molecular mechanism underlying biosynthesis and accumulation of these metabolites in peanut.

## Introduction

Cultivated peanut (*Arachis hypogaea* L.) is rich in protein, oil, and nutrients. Peanut is an important oilseed crop of Asia, Africa, and Americas (FAOSTAT 2020^1^). The edible portion of the peanut consists of both kernel and the seed coat also called as peanut testa (Christman et al., 2018). Previous studies reported that the peanut testa is rich in nutrients, including 12.3% protein, 16.6% oil, 2.8% ash, and approximately 140∼150 mg/g phenolic compounds (Nepote et al., 2007). Phenolic compounds are usually enriched on the exosphere of plant tissue to protect the internal cells. Peanut testa is rich in such phenolic compounds (Ma et al., 2014). In recent years, with the deepening of research on the important role of plant polyphenols in human health, more attentions have been paid to the nutritional value and function of peanut testa (Yu et al., 2006; Constanza et al., 2012; Liu et al., 2020; Oliveira et al., 2021).

Flavonoids and non-flavonoids are the major polyphenols in peanut testa. Flavonoids have the basic structure which consists of a C6-C3-C6 carbon skeleton comprising two 6-carbon benzene rings (rings A and B) linked by a 3-carbon heterocyclic ring (ring C) (Nabavi et al., 2020). Based on the degree of oxidation of the ring C and the number of methyl or hydroxyl groups on the rings A and B, flavonoids are mainly divided into 12 subgroups: flavanones, flavones, isoflavones, flavonols, anthocyanidins, proanthocyanidins, chalcones, dihydrochalcones, dihydroflavonols, coumarins, aurones, and phlobaphenes (Winkel-Shirley, 2001; Yu et al., 2005; Sasaki and Nakayama, 2015). With the diversity of molecular polymerization and modification (glycosylation, acylation, and others), there are more than 9,000 kinds of flavonoids present in higher plants (Tanaka et al., 2009; Noda et al., 2017; Sun et al., 2020). Non-flavonoids have the basic structural skeletons different from that of flavonoids, such as stilbenes (C6-C2-C6), phenolic acids (C6-C1), and hydroxycinnamic acids (C6-C3), all of which have dietary and medical significance. Different from the flavonoid pathway, the non-flavonoids are formed through the shikimic and phenylpropanoid pathways (Crozier et al., 2009). The scope of flavonoids and non-flavonoids is still controversial. For example, one view is that stilbene belongs to flavonoids. And resveratrol, an important member of stilbenes, is considered to have great potential in the treatment of cancer (Winkel-Shirley, 2001; Sirerol et al., 2016).

As one of the most important secondary metabolites in plants, flavonoids play an important role in plant growth, development and resistance to biotic, and abiotic stresses. Anthocyanidins are water-soluble pigments and important member of flavonoids in plants. They are important coloring substances of plant tissues, such as fruits and flowers with red, orange, blue, and purple colors (Grotewold, 2006; Dong et al., 2019), which can attract animals and insects, thus promoting pollination (Bradshaw and Schemske, 2003). Moreover, anthocyanidins have reactive oxygen species (ROS) scavenging ability and protect plants against damage from biotic and abiotic stresses (Cavaiuolo et al., 2013). Proanthocyanidins are the flavonoids with stronger antioxidant capacity than anthocyanidins, and their content is as high as 17% (w/w) of the dry weight of peanut testa (Karchesy and Hemingway, 1986; Park et al., 2011). Many other flavonoids, as phytoalexins or antioxidants, have properties similar to anthocyanidins and play important roles in resistance to drought stress, cold, ultraviolet radiations, and resistance to microorganisms, pathogens and insects (Iwashina, 2003; Pourcel et al., 2007; Zhang et al., 2020). Flavonoids can provide protection against oxidative stress, which has been implicated in some human diseases (Karadag et al., 2009). For example, flavonoid play a positive role in the treatment of atherosclerosis, diabetes mellitus, chronic inflammation, and some types of cancers in humans (Pinent et al., 2004; Osakabe and Yamagishi, 2009; Liu et al., 2020; Oliveira et al., 2021). Due to these bioactivities for flavonoids in peanut testa, attempts have been made to use them in some form as functional food ingredients or purified drug (Francisco and Resurreccion, 2009; Hathorn and Sanders, 2012; Christman et al., 2018). Flavonoids are generated from phenylalanine through the phenylpropanoid pathway, which has been clearly elucidated in model plants. With a series of enzyme reactions, metabolites flow to flavonols, flavanols, isoflavones, anthocyanidins and other branches, forming flavonoids substance with great differences in structure and content (Dong and Lin, 2021).

Testa color is an important qualitative trait of peanut. There are abundant phenotypic variations available in peanut testa color including pink, red, purple (black), white, tan, and multicolor, pink is most common in peanut germplasm. The composition and content of anthocyanidins are closely related to the color intensity of peanut testa. For instance, cyanidin-3-O-sambubioside which is a main anthocyanidin presents in black testa peanut cultivars (Kuang et al., 2017). With the completion of genome sequencing of cultivated peanut, great progress has been made in the research on the molecular mechanism of peanut seed coat color formation and gene identification. The candidate gene *AhTc1* that controls the color of peanut purple testa has been mapped on chromosome 10 and encodes for R2R3-MYB transcription factor (Zhao et al., 2019). Two independently inherited genes controlling peanut red testa, *AhRt1* and *AhRt2*, were identified on chromosomes 3 and 12, respectively (Chen et al., 2021; Zhang et al., 2022). Some other candidate genes and critical pathway were also predicted by combining transcriptome and metabolome methods between testa of two different colors, which enriched the study of gene function and the relationship between genes and metabolites in peanut testa (Wan et al., 2020; Xue et al., 2021). However, there are very few reports on the metabolomics studies of multiple testa colors of peanut. The metabolic pathways involved in flavonoid biosynthesis and molecular mechanism underlying testa color formation in peanut is still unclear.

Therefore, in this study, flavonoids content in various peanut testa color differences were investigated with the help of metabolomics profiling of four different colored peanut testas. The results provided insights on characteristics and direction of flavonoids accumulation in different colored peanut testa and supported for improving cultivated peanut with target color and flavonoid quality.

## Materials and methods

### Plant materials and treatments

The four peanut cultivars namely Yuhua 29 (YH29) with purple testa, Zhonghua 12 (ZH12) with red testa, Weihua 10 (WH10) with pink testa, Kainongbai (KNB) with white testa were planted at Jiyang Experimental Station of Shandong Academy of Agricultural Sciences (SAAS), Shandong, China (36°58′34.53″ N, 116°59′1.29″ E) during 2020. The peanut testa samples were peeled carefully and collected when the seeds were on the period of 70 days after pegging (DAP 70). Three biological replicates were performed for each peanut cultivar and each replicate contained the testa of 10 seeds from many plants of this variety. Then samples were rapidly frozen in liquid nitrogen and stored at −80°C.

### Chemicals and reagents

HPLC-grade methanol, water, acetonitrile was purchased from Thermo Scientific (Rockford, IL, USA), AR-grade chloroform was purchased from Titan (Shanghai, China), HPLC-grade flavonoid standards was purchased from Sigma-Aldrich (Shanghai) Trading Co., Ltd., (Shanghai, China) and Yuanye (Shanghai, China). All the other chemicals were of analytical grade.

### Sample extraction

The powdered sample (50 mg) was extracted with 600°μl of aqueous methanol (v:v = 1:2, containing succinic acid-2,2,3,3-d4, 50°ng/mL) using a rapid grinder (JXFSTPRP-24/32; Shanghai, China) at a frequency of 60 Hz for 2 min, followed by 20 min of sonication at 4°C (SB-5200DT, Ningbo, China). Subsequently, the samples were centrifuged at 13,000 rpm for 10 min at 4°C. 500°μl supernatant was transferred into new centrifuge tube. The residue added 400°μl methanol, extracted and centrifuged through the same steps as above, got 300°μl supernatant. The two extraction supernatants were mixed to obtain a total of 800°μl. Took 200°μl of supernatant and evaporated, then redissoluted with 200°μl aqueous methanol (v:v = 18:7, containing standard l-2-chlorophenylalanine, 10°ng/ml). Centrifuged at 13,000 rpm for 5 min at 4°C, the supernatants were filtered using 0.22-μm organic phase pinhole filter. Transfer them to brown LC injection vial and store at −80°C until LC-MS/MS analysis.

### Flavonoids-metabolites detection and multiple reaction monitoring

Metabolite quantification was analyzed by multiple reaction detection (MRM) mode of triple quadrupole mass spectrometry (MS). Flavonoids-metabolites identification and quantification was carried out by Shanghai Luming Biological Technology Co., Ltd., (Shanghai, China) using UPLC-ESI-MS/MS system (UPLC, AB ExionLC, Applied Biosystems Sciex; MS, Qtrap 6500+, Applied Biosystems Sciex). The analytical conditions were as follows: HPLC: column, Waters UPLC HSS T3 (100*2.1 mm, 1.8 μm); solvent system, mobile phase A (0.01% formic acid): mobile phase B (acetonitrile); gradient program, 100:0 V/V at 0°min, 5:95 V/V at 11.0 min, 5:95 V/V at 12.0 min, 95:5 V/V at 12.1 min, 95:5 V/V at 15.0 min; flow rate, 0.40 mL/min; temperature, 40°C; injection volume: 5°μL. The effluent was alternatively connected to an ESI-triple quadrupole-linear ion trap (Q TRAP)-MS system. Linear ion trap (LIT) and triple quadrupole (QQQ) scans were acquired on an API 4500 Q TRAP LC/MS/MS system, equipped with an ESI Turbo Ion-Spray interface, analyst software (AB Sciex, version 1.6) was used for instrument control, data acquisition, and subsequent quantification. A combination of both ionization modes (positive and negative) in MS full scan mode was applied for the molecular mass determination of the compounds in samples of peanut testa. The MS conditions for positive ionization modes optimized are as follows: the column oven was set at 35°C; curtain gas, 30°psi; ion spray voltage, 5,500 V; temperature, 600°C; ion source gas 1, 60°psi; and ion source gas 2, 50°psi. The MS conditions for negative ionization modes are as follows: the column oven was set at 35°C; curtain gas, 30°psi; ion spray voltage, −4,500 V; temperature, 600°C; ion source gas 1, 60°psi; and ion source gas 2, 50°psi. QQQ scans were acquired during MRM experiments with collision gas (nitrogen) set to 5°psi. DP and CE for individual MRM transitions were done with further DP and CE optimization. A specific set of MRM transitions was monitored for each period according to the metabolites eluted within the period. The MRM for each cultivar was performed in triplicate. Three technical replicates per sample were also analyzed in each series to assess technical reproducibility (Quality Controls: QCs). Through the overlapping display analysis of the total ions current (TIC) of MS detection and analysis of quality controls (QCs), the average variation in metabolite abundance within the QCs (RSD) was < 20%. It shows that the signal stability of LC-MS/MS in different time periods is good.

### Statistical analysis

All experiments were performed in triplicates and the results were presented as mean ± standard deviation. Student’s *t*-test and fold change analysis were used to compare the differential metabolites between the two groups. The significant differences [*p* < 0.05 and | log2(FC) | > 1] among the mean values of different samples were analyzed by performing the Duncan test using IBM SPSS Statistics version 25 (IBM Corporation, New York, NY, USA). Correlation analysis used Pearson correlation coefficient, which measured the degree of linear correlation between two quantitative variables. The KEGG ID of different metabolites was used for pathway enrichment analysis to obtain the enrichment results of metabolic pathways.

## Results and discussion

### Phenotypic and extractive differences among the four peanut germplasms

**FIGURE 1:**
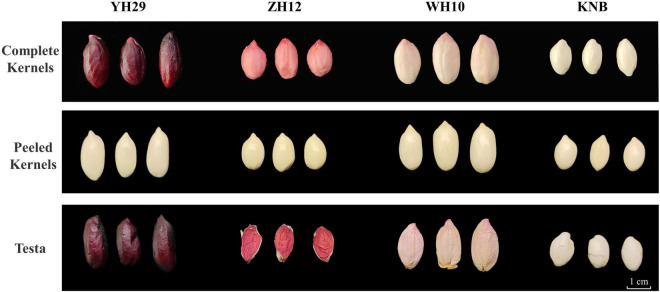
Phenotypic differences in the four color testa of peanut. Differences of complete kernels, peeled kernels, and testa color phenotypes among four varieties on the period of DAP 70.

The four peanut cultivars including Yuhua29 (YH29), Zhonghua12 (ZH12), Weihua10 (WH10), and Kainongbai (KNB) showed purple, red, pink, and white, testa colors respectively (Figure 1). The variation of metabolic components in these four peanut genotypes with different testa colors was investigated using targeted metabolomics analysis. The target metabolites were detected qualitatively and quantitatively by the method of UPLC-ESI-MS/MS (Ultra performance liquid chromatography-electrospray ionization-tandem mass spectrometry). Clear quantitative and qualitative differences were revealed with a first inspection of the raw data plotted as TIC (Total ions current) and base peak chromatograms, respectively (Supplementary Figure 1A). Raw data was processed in a target manner based on the dedicated analysis package which included absolute quantitative data of 130 phenolic substances in 13 categories, namely anthocyanidins, flavones, flavonols, flavanones, isoflavones, coumarins, dihydrochalcones, phenylpropanoids, proanthocyanidins, benzoic acid derivatives, catechin derivatives, stilbenes, and terpenoids. Most of the above substances were belong to flavonoids, and the rest were polyphenols closely related to flavonoids. A total of 85 metabolites were identified in four peanuts (Supplementary Table 1). These identified metabolites belonged to different subgroups, including 9 phenylpropanoids, 13 benzoic acid derivatives, 7 catechin derivatives, 3 stilbenes, 2 dihydrochalcones, 5 anthocyanidins, 5 flavones, 16 flavonols, 9 flavanones, 2 terpenoids, 4 coumarins, 6 isoflavones, and 4 proanthocyanidins.

**FIGURE 2:**
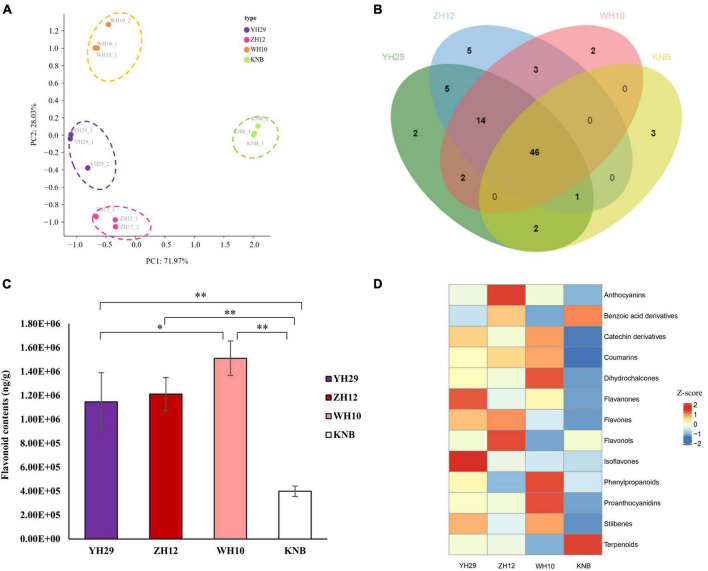
Extractive composition differences in type and content among the four samples. **(A)** Principal component analysis (PCA) score plots for metabolites in four types of peanut testa based on LC-MS/MS. **(B)** Distribution of the 85 flavonoids in the different colored testa. **(C)** The total content of flavonoids detected in the four samples. **(D)** The comparison of the relative contents of different type of flavonoids in the four samples. Row data is normalized into Z-score.

Principal Component Analysis (PCA) confirmed that the biological replicates of each sample were well-correlated. Moreover, there were obvious differences between the four testa with different colors, which also demonstrated the different metabolite composition among the four samples (Figure 2A). We found that 46 metabolites were present in all four samples, and 2, 5, 2, and 3 metabolites were specific in YH29, ZH12, WH10, and KNB, respectively (Figure 2B).

We found that the total content of flavonoids was highest in the pink testa of WH10, and lowest in the white testa of KNB (Figure 2C). The absolute content of various types of flavonoids revealed that proanthocyanidins, flavourols, catechin derivatives, phylpropanoids, and anthocyanins were the main flavonoids in peanut testa, while proanthocyanidins contributed more than half of the total flavonoids in the pink which contained the most flavonoids in four samples (Supplementary Figure 1B). The comparison of the relative contents of different groups of flavonoids in the four samples showed that the isoflavones and flavanones were abundant in YH29, anthocyanins, and flavourols were abundant in ZH12, phenopropanoids, proanthocyanidins, and dihydrochalcones were abundant in WH10, and terpenoids and benzoic acid derivatives were abundant in KNB, respectively (Figure 2D).

### Distinguished metabolites in four peanut testa samples with different colors

**FIGURE 3:**
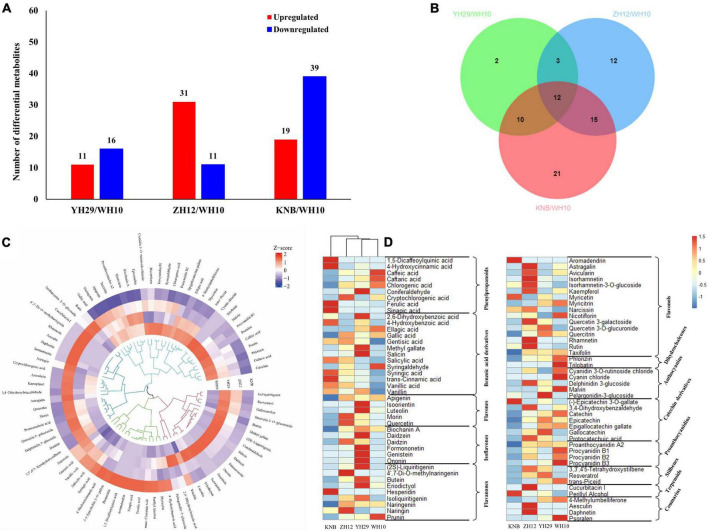
Statistics of distinguished metabolites in four peanut testa samples with different colors. **(A)** Differential accumulated metabolites (DAMs) between WH10 of the most common pink testa and other three. The blue color represents down-regulated metabolites, and the red color represents up-regulated metabolites. The significant difference conforms to *p* < 0.05 and | log2(FC) | > 1. **(B)** Venn diagram exhibiting the number of the key flavonoids related to the pink testa color. **(C)** The circular plot of hierarchical cluster analysis illustrating the classification of flavonoids. **(D)** The heatmap of differential metabolite divided into 13 subgroups. Row data is normalized into Z-score.

In total, 78 differentially accumulated metabolites (DAMs) were identified across four testa colors (Supplementary Figure 2). Among them, 64 DAMs were differentially accumulated between the red (ZH12) and white (KNB) peanut testa colors, including 52 up-regulated and 12 down-regulated flavonoids (Supplementary Figure 3A). However, only 27 differentially expressed flavonoids were identified between purple (YH29) and pink (WH10) peanut testa colors (Figure 3A). We also observed that there was more kind of flavonoids in red testa peanut (ZH12) which had a total of 74 kinds, while there were only 52 kinds of flavonoids were identified in white (KNB) testa.

To find the key flavonoids in the formation of pink testa, Venn analysis was performed among the differential metabolites between YH29 and WH10, ZH12 and WH10, and KNB, and WH10 (Figure 3B). The result revealed that there were 12 overlapping significantly differential flavonoids among the three pairwise comparisons. Similarly, we compared the YH29, ZH12, and KNB against the other testa and detected 10, 15, and 30 overlapping differential metabolites, respectively (Supplementary Figure 3). In total, 49 overlapping different flavonoids were obtained (Table 1). These overlapping flavonoids may play an important role in the development of different colors of peanut testa.

**TABLE 1:** Key 49 significantly differential metabolites between the different colored samples.

Class	Compounds	Samples	KEGG ID
Anthocyanins	Cyanidin 3-O-rutinoside chloride	YH29 KNB	–
Anthocyanins	Cyanin chloride	WH10	–
Anthocyanins	Malvin	YH29 WH10	–
Anthocyanins	Pelargonidin-3-glucoside	YH29 KNB	C12137
Benzoic acid derivatives	Gentisic acid	WH10	C00628
Benzoic acid derivatives	Methyl gallate	YH29 ZH12	–
Benzoic acid derivatives	Salicin	YH29	C01451
Catechin derivatives	3,4-Dihydroxybenzaldehyde	WH10 YH29 ZH12 KNB	C16700
Catechin derivatives	Catechin	KNB	C06562
Catechin derivatives	Epicatechin	KNB	C09727
Catechin derivatives	Protocatechuic acid	WH10 KNB	C00230
Coumarins	4-Methylumbelliferone	ZH12 KNB	C03081
Coumarins	Aesculin	ZH12	C09264
Coumarins	Daphnetin	ZH12	C03093
Dihydrochalcones	Phlorizin	KNB	C01604
Dihydrochalcones	Trilobatin	WH10	–
Flavanones	4’,7-Di-*O*-methylnaringenin	ZH12	–
Flavanones	Eriodictyol	KNB	C05631
Flavanones	Hesperidin	KNB	C09755
Flavanones	Naringenin	KNB	C00509
Flavanones	Prunin	WH10 KNB	–
Flavones	Apigenin	KNB	C01477
Flavones	Morin	KNB	C10105
Flavones	Quercetin	KNB	C00389
Flavonols	Aromadendrin	KNB	C00974
Flavonols	Astragalin	ZH12	C12249
Flavonols	Isorhamnetin	ZH12	C10084
Flavonols	Isorhamnetin-3-O-glucoside	ZH12 WH10	–
Flavonols	Kaempferol	ZH12 WH10	C05903
Flavonols	Nicotiflorin	YH29	–
Flavonols	Quercetin 3-galactoside	ZH12	C10073
Flavonols	Quercetin 3-O-glucuronide	WH10 KNB	–
Flavonols	Rhamnetin	ZH12	–
Flavonols	Rutin	ZH12	C05625
Flavonols	Taxifolin	KNB	–
Isoflavones	Genistein	YH29	C06563
Phenylpropanoids	1,5-Dicaffeoylquinic acid	KNB	C10445
Phenylpropanoids	4-Hydroxycinnamic acid	KNB	C00811
Phenylpropanoids	Caffeic acid	WH10 KNB	C01481
Phenylpropanoids	Caftaric acid	KNB	–
Phenylpropanoids	Chlorogenic acid	KNB	C00852
Phenylpropanoids	Ferulic acid	KNB	C01494
Phenylpropanoids	Sinapic acid	KNB	C00482
Proanthocyanidins	Proanthocyanidin A2	KNB	C10237
Proanthocyanidins	Procyanidin B1	YH29 KNB	–
Proanthocyanidins	Procyanidin B2	YH29 KNB	C17639
Proanthocyanidins	Procyanidin B3	WH10 KNB	–
Stilbenes	trans-Piceid	ZH12 KNB	C10275
Terpenoids	Cucurbitacin I	ZH12	C08800

In order to visualize the accumulation patterns of metabolites in peanut testa with different colors, multivariate data analysis was performed (Figure 3C). Many specific metabolites were identified in different peanut testa colors, suggesting that the testa tissues might different metabolic pathways synthesizing different pigments. In addition, we also found that each testa had higher accumulation of specific metabolites as compare to other testa colors, implying different metabolic directions of flavonoids existing in differently colored testa of peanuts (Figure 3D). For instance, the phenylpropanoids and benzoic acid derivatives content were higher in white testa (KNB) than other three peanut testa colors. Moreover, the content of secondary metabolites in white testa of KNB after entering in the flavonoid pathway was significantly lower than that of the other samples. Some of the ultimate products like anthocyanidins and proanthocyanidins were not detected in KNB, indicating that the metabolites of white testa of KNB did not enter in the flavonoid production after phenylalanine biosynthesis pathway which is primary stage of flavonoid production.

Moreover, we found that the red testa of ZH12 had a higher content of flavonols. The purple testa of YH29 had a high level of flavonoids, flavanones and isoflavones, while pink testa of WH10 contained more downstream metabolites, such as dihydrochalcones, catechin derivatives, and proanthocyanidins (Figure 3D). Recently, the proanthocyanidins extracted from peanut testa were identified as a novel allosteric AKT inhibitor with potent anti-tumor efficacy beyond its antioxidant and anti-inflammatory properties (Liu et al., 2020). Here, we also observed that the total amount and types of proanthocyanidins which are terminal metabolites in flavonoid biosynthesis pathway were significantly higher in pink testa as compare to other peanut testa colors.

**FIGURE 4:**
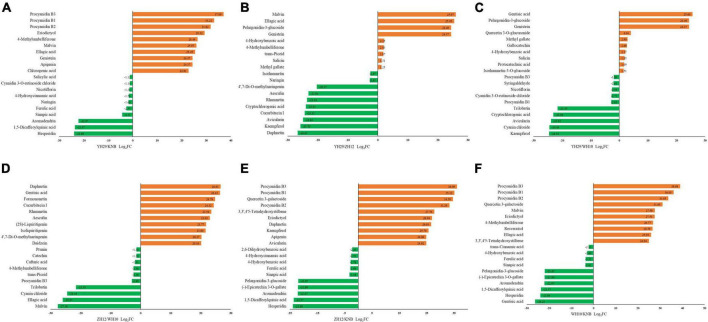
Top 20 differentially accumulated metabolites (DAMs) among the six pairwise groups of the fold change. **(A)** YH29 and KNB; **(B)** YH29 and ZH12; **(C)** YH29 and WH10; **(D)** ZH12 and WH10; **(E)** ZH12 and KNB; and **(F)** WH10 and KNB.

To better understand the variation of metabolic components between different peanut testa colors, we performed a pairwise comparison and identified top 20 various significant metabolites (Figure 4). The procyanidins content in pink testa (WH10) was significantly higher as compare to other testa colors. The purple testa (YH29) consistently showed higher anthocyanidin content. The red testa (ZH12) contained more coumarins and flavonols, such as daphnetin, aesculetin, kaempferol and avicularin. Meanwhile, phenylpropanoid, and benzoic acid derivatives were higher in white testa (KNB), for example 4-hydroxybenzoic acid, sinapic acid, 4-hydroxycinnamic acid, and ferulic acid.

An increasing amount of evidence has showed that many flavonoids had biological activities, and the functions of flavonoids in nutrition and health care have been excavated (Supplementary Table 2). For example, the hydroxyl and carbonyl groups in the structure of most of the flavonoids could avoid oxidative damage by preventing the production of free radicals and scavenging their antioxidant activity (Rice-evans et al., 1995; Burda and Oleszek, 2001). Along with the antioxidant function, some flavonoids had anticancer (Imran et al., 2019), antitumor (Selvendiran et al., 2006), anti-inflammatory (Lee et al., 2011), antibacterial (Comalada et al., 2005), analgesic (Khan, 2017), antiallergic (Baghel et al., 2012), and many other health benefits. In addition, some compounds could also be used as nutritional additives, spices or essence (Raskin, 1992; Suresh et al., 2009). We can use these findings to deploy targeted and precise breeding of peanuts, and develop peanut varieties with high-quality, high content of target metabolites to develop the peanuts as functional foods.

### Correlation analysis of differential metabolites in peanut testa

**FIGURE 5:**
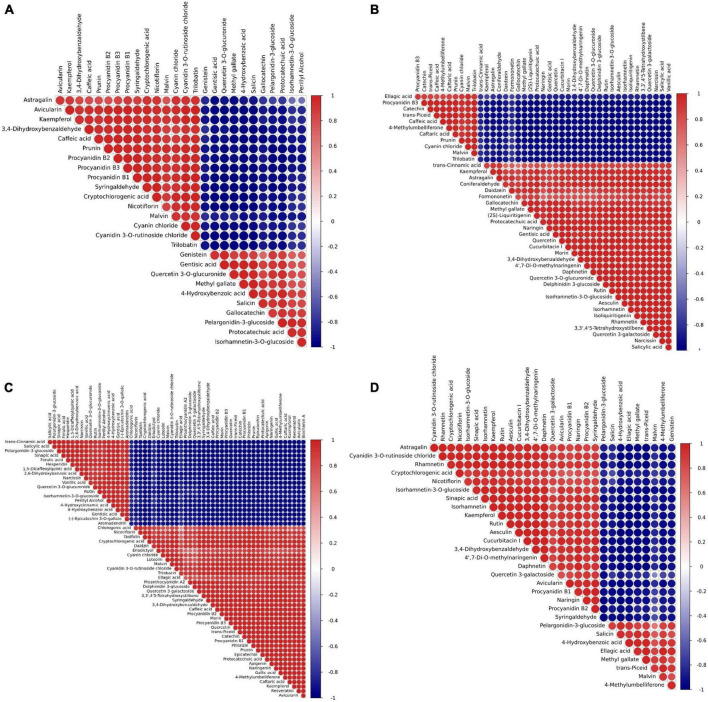
Pearson correlation analysis results for differential metabolites by pairwise. **(A)** YH29 and WH10; **(B)** ZH12 and WH10; **(C)** WH10 and KNB; and **(D)** YH29 and ZH12. Dark red represents a significant positive correlation. Dark blue represents a significant negative correlation.

Since there are diverse metabolites present in various colors of peanut testa, it is an interesting to analyze the association between different metabolic compounds. Pearson correlation analysis showed significant (*p* < 0.05) pairwise correlation based on 26 flavonoids between purple (YH29)/pink (WH10) testa colors (Figure 5A). While, pairwise correlation based on 41 flavonoids between red testa (ZH12)/pink testa (WH10), and 57 flavonoids between pink testa (WH10)/white (KNB) (Figures 5B,C). The performance of some flavonoids in different correlation comparison groups was consistent. For instance, proanthocyanidins B1, B2, and B3 in pairs were consistently showed positive correlation. Proanthocyanidins were positively correlated with cyanidin 3-O-rutinoside and malvin, but negatively correlated with pelargonidin-3-glucoside (Figure 5D). Interestingly, these are all anthocyanidins. There were also some flavonoids with different correlations in different groups. For instance, proanthocyanidins were positively correlated with kaempferol and astragalin in YH29/WH10, WH10/KNB, YH29/ZH12, and ZH12/KNB, while negatively correlated in ZH12/WH10 (Supplementary Figure 4). These results indicated that the flavonoid metabolic pathway is basically same in different testa color varieties, however some changes might occur due to individual gene variations in the pathway.

### Enrichment analysis of metabolic pathways and flow direction of metabolites

**FIGURE 6:**
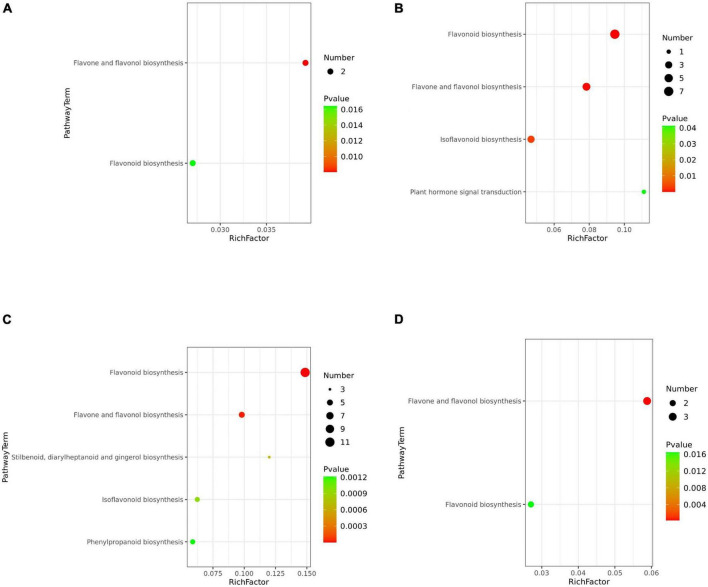
Bubble plot of KEGG metabolic pathway enrichment analysis. **(A)** YH29 and WH10; **(B)** ZH12 and WH10; **(C)** WH10 and KNB; and **(D)** YH29 and ZH12. The *p*-value in the metabolic pathway is the significance of the enrichment of the metabolic pathway, and the significant enrichment pathway is selected for bubble plot. The ordinate is the name of metabolic pathway. The abscissa is the enrichment factor (rich factor means number of significantly different metabolites/number of total metabolites in the pathway). The larger the rich factor, the greater the enrichment degree. The color from red to green indicates that *p*-value decreases in turn. The larger the point, the more metabolites enriched on the pathway.

In the pink testa peanut WH10, the pathways of “Flavone and flavonol biosynthesis” and “Flavonoid biosynthesis” were highly enriched (Figure 6A). Flavonoid biosynthesis, flavone and flavonol biosynthesis were significantly differential metabolites between red (ZH12) and pink (WH10) were mostly involved in flavonoid biosynthesis, (Figure 6B). The differential metabolites between white (KNB) and pink (WH10) were involved flavonoid biosynthesis, flavone and flavonol biosynthesis (Figure 6C). Our data indicated that the differential metabolites between YH29 and ZH12 were enriched mainly in flavone and flavonol biosynthesis (Figure 6D). The KEGG between YH29/KNB and ZH12/KNB enriched mainly in flavonoid biosynthesis, flavone and flavonol biosynthesis, isoflavonoid biosynthesis, and phenylpropanoid biosynthesis (Supplementary Figure 5).

**FIGURE 7:**
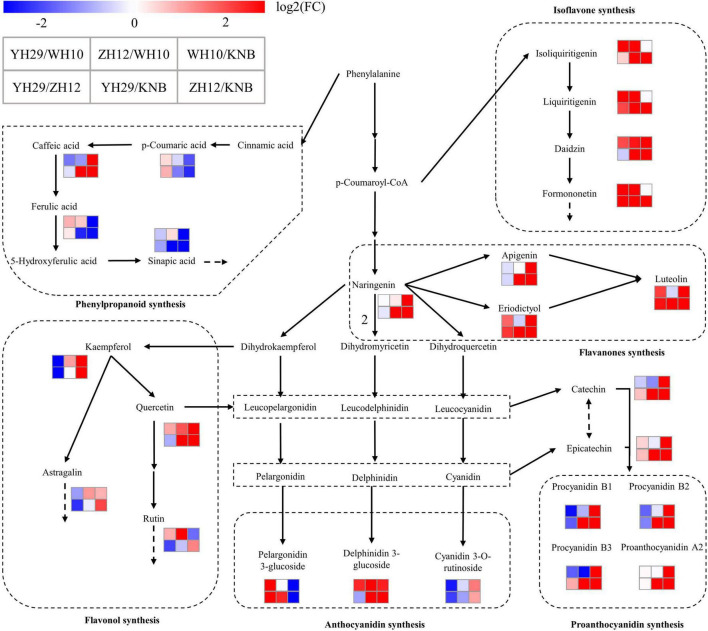
Schematic diagram of the metabolic pathway and relative content of metabolites. **(A)** ZH12 and WH10; **(B)** WH10 and KNB. The absolute content is shown in the small histogram next to the compound using the average of all values. The metabolic pathway was modified based on the KEGG database (http://www.genome.jp/kegg/).

A schematic diagram was deduced to illustrate the selected differential metabolites among the six pairwise comparisons in the flavonoid biosynthesis pathway (Figure 7). Compared with the pink (WH10), the flavonoids in the testa of red (ZH12) obviously flowed to the isoflavone, flavonol and anthocyanin synthesis pathways. In contrast, the content of proanthocyanidin and catechin, which was the proanthocyanidin synthetic monomers, in WH10 is significantly higher than that in ZH12. Therefore, we concluded that there are several “key nodes” or “switches” in the pink and red testa peanut varieties, which controlled the accumulation and flow direction of flavonoids which results in significant differences in flavonoids metabolites in testa of red and pink color. We speculated that the most likely “key nodes” between red (ZH12) and pink (WH10) were the branches from the main flavonoid biosynthesis pathway to the isoflavone pathway, to the flavonol pathway and to the proanthocyanidin pathway.

The obviously different flavonoid accumulation patterns were also observed between pink (WH10) and white (KNB) testa colors (Figure 7). The compounds in the middle and downstream of flavonoid synthesis pathway accumulated significantly in pink (WH10) testa, while in white (KNB) testa, the derivative content of phenylalanine, which were the flavonoid pathway starting substance, was much higher than that in pink (WH10) testa. The content of naringin in pink peanut was also much higher compare to white testa peanut, we speculated that this “key nodes” should be before the synthesis of naringin, or even at the initiation of phenylalanine biosynthesis.

In previous studies, some key genes have been found to regulate anthocyanin accumulation in peanut testa. The candidate gene controlling peanut purple testa was considered to be one of two MYB transcription factors (Zhao et al., 2019; Huang et al., 2020). Two independently inherited genes might be controlling peanut red testa, One encoded bHLH transcription factor and the other encoded anthocyanin reductase (Chen et al., 2021; Zhang et al., 2022). By analyzing the pink and red testa of peanut by transcriptome and metabolome, some CHS, F3′H, DFR, MYB, bHLH, and WD40 genes may be the key formation and regulatory genes controlling the formation of pink and red testa in peanut (Xue et al., 2021). FLS, DFR, and WD40 transcription factor gene might be the key responsible for the white testa phenotype (Wan et al., 2020). Combined with our research, these key genes may be the structural genes at the node, or regulatory factors (for example, some transcription factors) acting at the node, which the changes of these might be the root cause behind the difference in metabolite accumulation in various peanut testa colors. Our study is helpful to determine the function of these genes and the molecular mechanism of regulation.

## Conclusion

In this study, we performed accurately targeted metabolite profiling by UPLC-MS/MS of four types peanut testa colors to evaluate the metabolite differences. We observed that the different accumulation patterns of flavonoids in peanut testa of different colors are related to the flow of flavonoid metabolites across the pathways. The different effects of structural genes or regulatory factors near nodes in different testa color peanuts might lead to the final flavonoid metabolome differences. Moreover, we also discussed the nutritional and health care effects of flavonoids, and the prospect of breeding of peanut varieties high flavonoids content.

## Data availability statement

The original contributions presented in this study are included in the article/Supplementary material, further inquiries can be directed to the corresponding authors.

## Author contributions

YZ and CZ conceived the project. KZ, JM, SG, LH, HX, NL, JP, RT, and HH carried out the experiments and analyzed the experimental results. KZ and JM wrote the original draft. CZ, XW, YZ, and SG helped to review and revise the original draft. All authors have read and approved the final manuscript.

## Footnotes

1
http://www.fao.org/faostat/en/#data/

## Supplementary material

Supplementary Figure 1 Extractive composition differences in type and content among the four samples. **(A)** The total ions current (TIC) of the samples. The abscissa is the retention time (RT) of the metabolite detection, and the ordinate is the ionic strength (count per second, CPS) of the ion detection. **(B)** The absolute content of various types of flavonoids.

Supplementary Figure 2 Volcano plots of differential metabolites in the pairwise comparison between: **(A)** YH29 and KNB; **(B)** YH29 and ZH12; **(C)** YH29 and WH10; **(D)** ZH12 and WH10; **(E)** ZH12 and KNB; and **(F)** WH10 and KNB. Each point in the volcano map represents a metabolite, the abscissa represents the logarithmic value of the difference of relative content of a certain metabolite in the two samples, the ordinate represents the VIP value. The greater the absolute value of the abscissa, the greater the multiple difference in the expression level between the two samples; the greater the ordinate value, the more significant the differential expression, and the more reliable the differentially expressed metabolites screened. In the figure, the blue dots represent down-regulated differentially metabolites, the red dots represent up-regulated differentially metabolites, and gray represents detected but not significantly different metabolites.

Supplementary Figure 3 Statistics of distinguished metabolites in four peanut testa samples with different colors. **(A)** Differential accumulated metabolites (DAMs) among YH29/KNB, ZH12/KNB, and YH29/ZH12. The blue color represents down-regulated metabolites, and the red color represents up-regulated metabolites. The significant difference conforms to *p* < 0.05 and | log2(FC) | > 1. Venn diagram exhibiting the number of the key flavonoids related to **(B)** YH29; **(C)** ZH12; and **(D)** KNB.

Supplementary Figure 4 Pearson correlation analysis results for differential metabolites by pairwise. **(A)** YH29 and KNB; **(B)** ZH12 and KNB. Dark red represents a significant positive correlation. Dark blue represents a significant negative correlation.

Supplementary Figure 5 Bubble plot of KEGG metabolic pathway enrichment analysis. **(A)** YH29 and KNB; **(B)** ZH12 and KNB. The *p*-value in the metabolic pathway is the significance of the enrichment of the metabolic pathway, and the significant enrichment pathway is selected for bubble plot. The ordinate is the name of metabolic pathway. The abscissa is the enrichment factor (rich factor means number of significantly different metabolites/number of total metabolites in the pathway). The larger the rich factor, the greater the enrichment degree. The color from red to green indicates that *p*-value decreases in turn. The larger the point, the more metabolites enriched on the pathway.
