# Supplementary figures and images for: Targeted metabolome analysis reveals accumulation of metabolites in testa of four peanut germplasms
Source: Front Plant Sci. 2022 Sep 16;13:992124. doi: 10.3389/fpls.2022.992124 (PMC9523574; doi:10.3389/fpls.2022.992124)

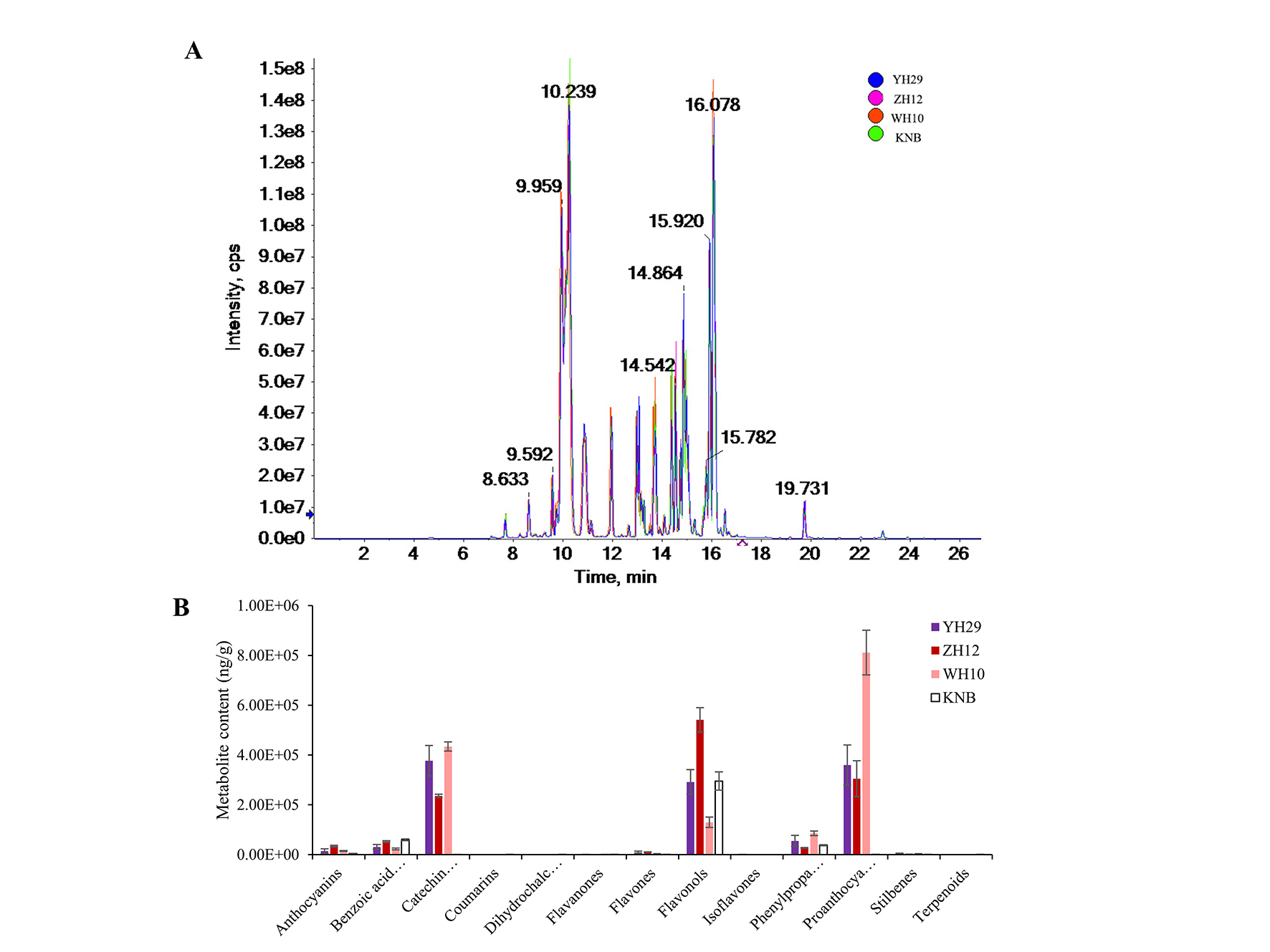

Supplement: Supplementary Figure 1 — Extractive composition differences in type and content among the four samples. (A) The total ions current (TIC) of the samples. The abscissa is the retention time (RT) of the metabolite detection, and the ordinate is the ionic strength (count per second, CPS) of the ion detection. (B) The absolute content of various types of flavonoids. [file Image_1.JPEG]

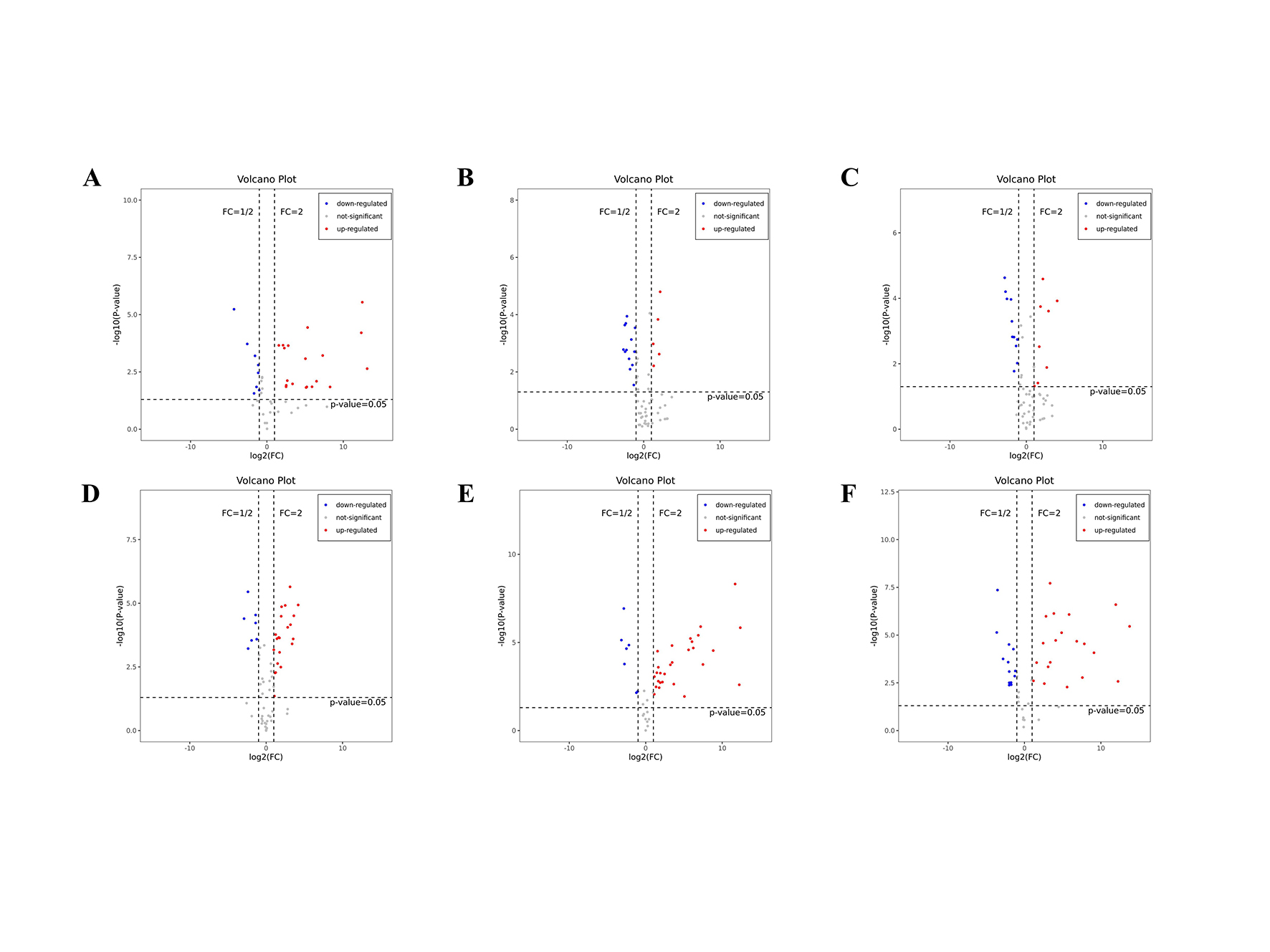

Supplement: Supplementary Figure 2 — Volcano plots of differential metabolites in the pairwise comparison between: (A) YH29 and KNB; (B) YH29 and ZH12; (C) YH29 and WH10; (D) ZH12 and WH10; (E) ZH12 and KNB; and (F) WH10 and KNB. Each point in the volcano map represents a metabolite, the abscissa represents the logarithmic value of the difference of relative content of a certain metabolite in the two samples, the ordinate represents the VIP value. The greater the absolute value of the abscissa, the greater the multiple difference in the expression level between the two samples; the greater the ordinate value, the more significant the differential expression, and the more reliable the differentially expressed metabolites screened. In the figure, the blue dots represent down-regulated differentially metabolites, the red dots represent up-regulated differentially metabolites, and gray represents detected but not significantly different metabolites. [file Image_2.JPEG]

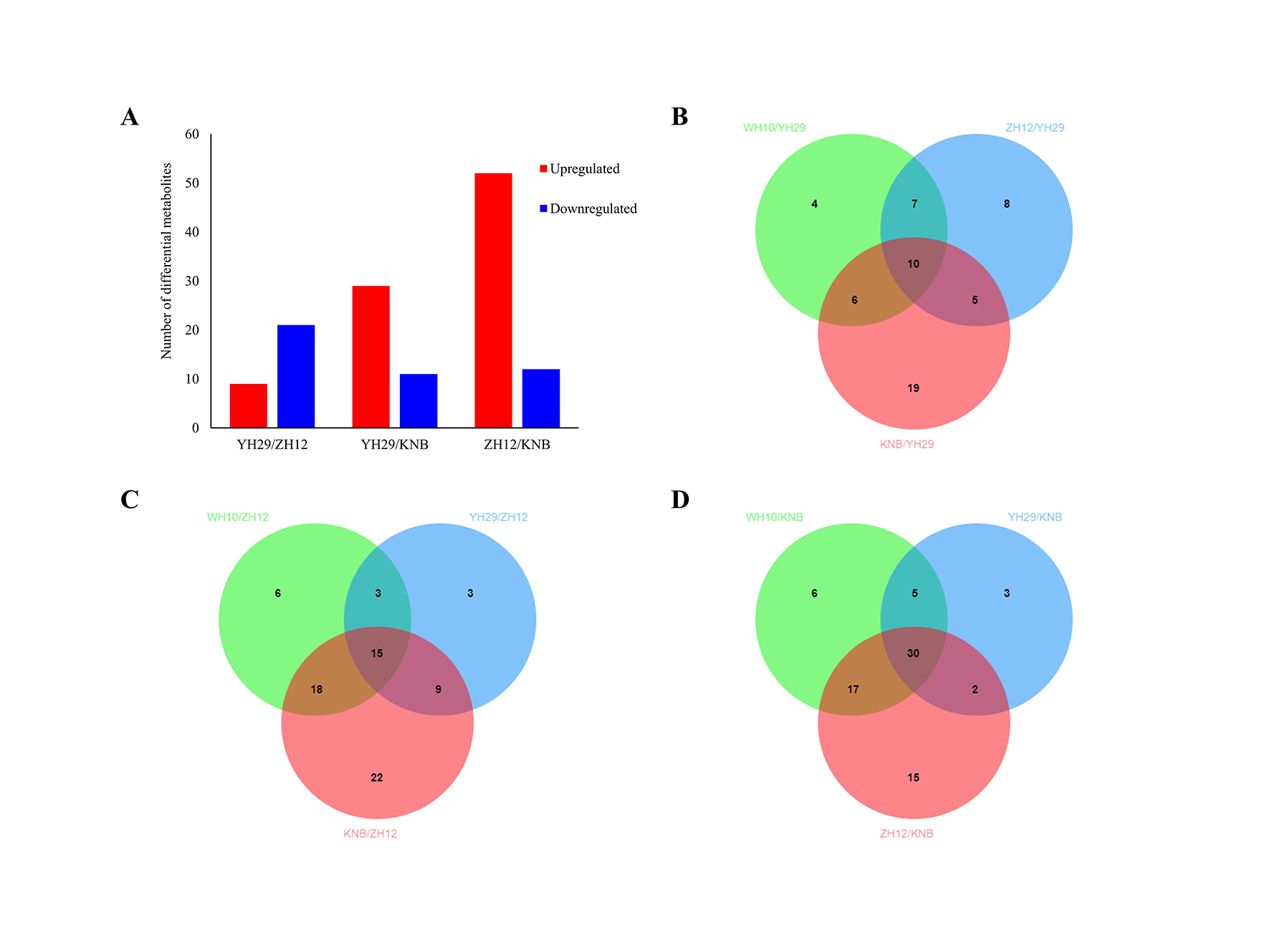

Supplement: Supplementary Figure 3 — Statistics of distinguished metabolites in four peanut testa samples with different colors. (A) Differential accumulated metabolites (DAMs) among YH29/KNB, ZH12/KNB, and YH29/ZH12. The blue color represents down-regulated metabolites, and the red color represents up-regulated metabolites. The significant difference conforms to p < 0.05 and | log2(FC) | > 1. Venn diagram exhibiting the number of the key flavonoids related to (B) YH29; (C) ZH12; and (D) KNB. [file Image_3.JPEG]

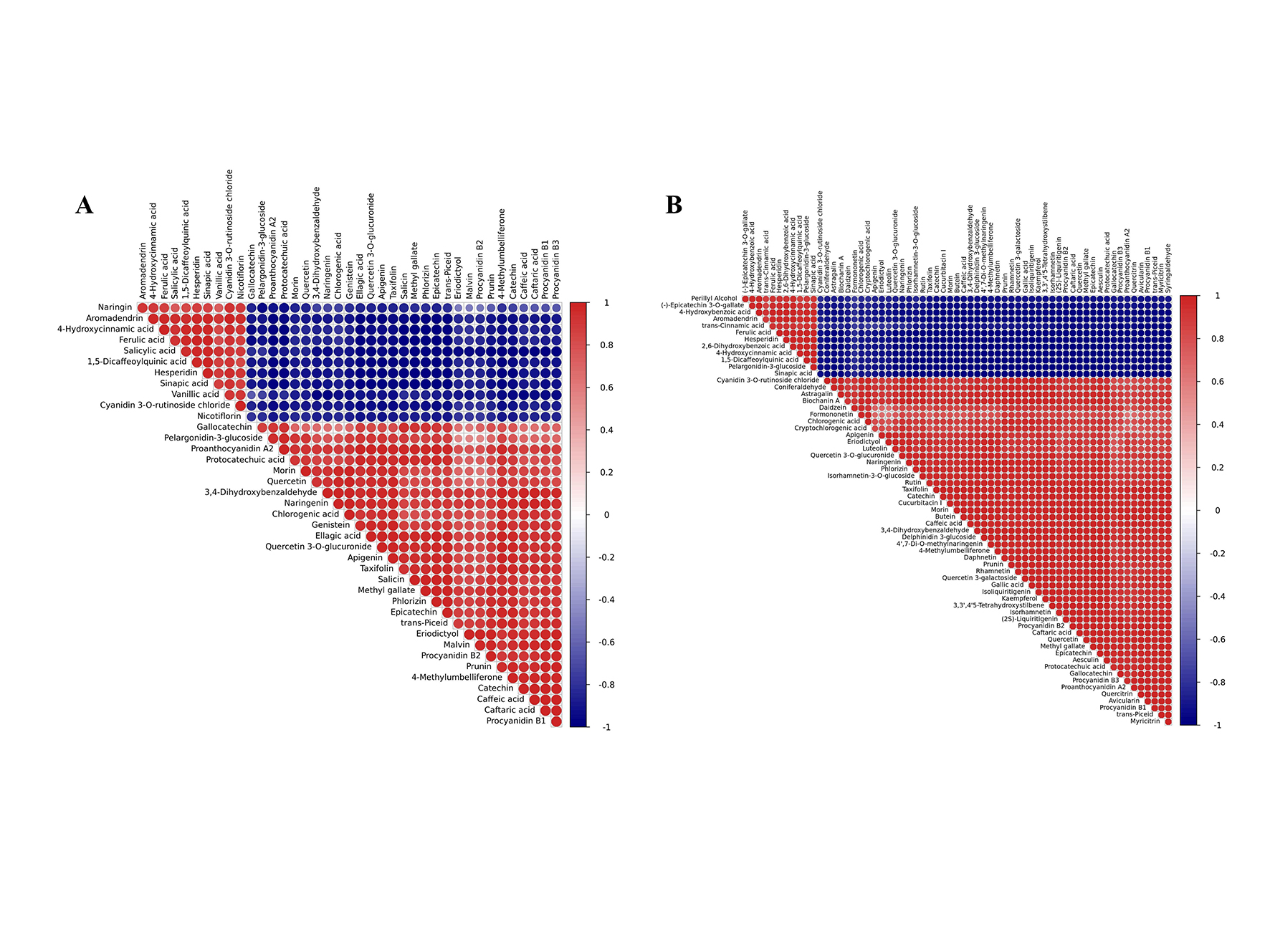

Supplement: Supplementary Figure 4 — Pearson correlation analysis results for differential metabolites by pairwise. (A) YH29 and KNB; (B) ZH12 and KNB. Dark red represents a significant positive correlation. Dark blue represents a significant negative correlation. [file Image_4.JPEG]

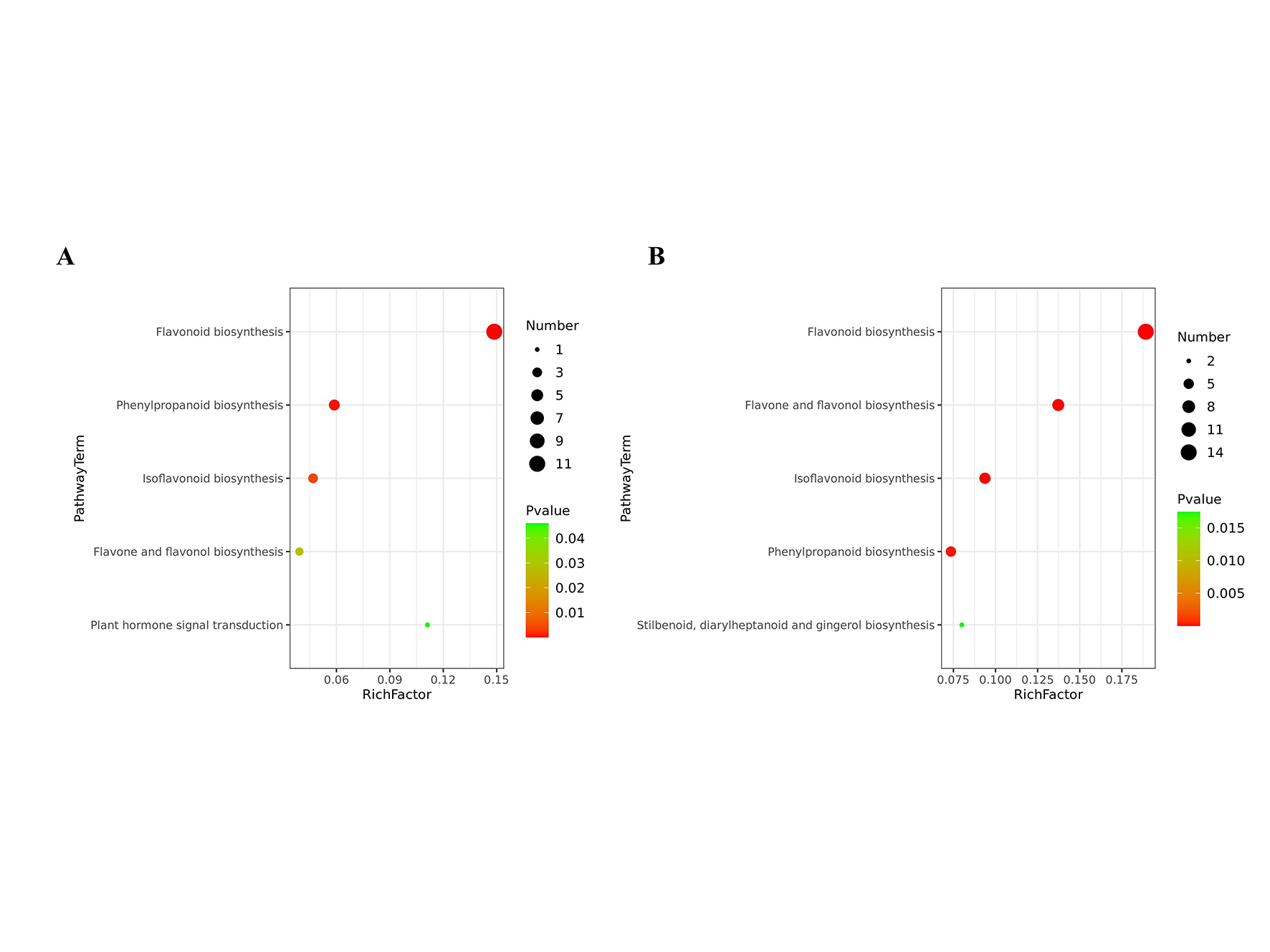

Supplement: Supplementary Figure 5 — Bubble plot of KEGG metabolic pathway enrichment analysis. (A) YH29 and KNB; (B) ZH12 and KNB. The p-value in the metabolic pathway is the significance of the enrichment of the metabolic pathway, and the significant enrichment pathway is selected for bubble plot. The ordinate is the name of metabolic pathway. The abscissa is the enrichment factor (rich factor means number of significantly different metabolites/number of total metabolites in the pathway). The larger the rich factor, the greater the enrichment degree. The color from red to green indicates that p-value decreases in turn. The larger the point, the more metabolites enriched on the pathway. [file Image_5.JPEG]
